# Gut Microbiota Modulation Through Mediterranean Diet Foods: Implications for Human Health

**DOI:** 10.3390/nu17060948

**Published:** 2025-03-08

**Authors:** Pasquale Perrone, Stefania D’Angelo

**Affiliations:** Department of Medical, Movement, and Wellbeing Sciences, Parthenope University of Naples, 80133 Naples, Italy; pasquale.perrone@collaboratore.uniparthenope.it

**Keywords:** antioxidants, cardiovascular diseases, extra virgin olive oil, gut microbiota, Mediterranean diet, polyphenols, short-chain fatty acids

## Abstract

The Mediterranean diet (MD) is widely recognized for its health benefits, particularly in modulating gut microbiota composition and reducing the risk of metabolic, cardiovascular, and neurodegenerative diseases. Characterized by a high intake of plant-based foods, monounsaturated fats, and polyphenols, primarily from extra virgin olive oil, the MD fosters the growth of beneficial gut bacteria such as *Bifidobacterium*, *Faecalibacterium prausnitzii*, and *Roseburia*, which produce short-chain fatty acids that enhance gut barrier integrity, reduce inflammation, and improve metabolic homeostasis. Clinical and preclinical studies have proved that the MD is associated with increased microbial diversity, reduced pro-inflammatory bacteria, and improved markers of insulin sensitivity, lipid metabolism, and cognitive function. Additionally, the MD positively influences the gut microbiota in various conditions, including obesity, cardiovascular disease, and neurodegeneration, potentially mitigating systemic inflammation and enhancing neuroprotective mechanisms. Emerging evidence suggests that MD variants, such as the Green-MD, and their integration with probiotics can further optimize gut microbiota composition and metabolic parameters. While the beneficial impact of the MD on the gut microbiota and overall health is well supported, further long-term clinical trials are needed to better understand individual variability and improve dietary interventions tailored to different populations.

## 1. Introduction

Chronic non-communicable diseases have become one of the leading causes of morbidity and mortality worldwide, placing a significant burden on healthcare systems and reducing the quality of life for millions of people. Among these, cardiovascular diseases, type 2 diabetes, neurological disorders, obesity, and metabolic syndromes are experiencing a steady increase in prevalence, particularly in industrialized countries. Several risk factors contribute to their onset, including sedentary lifestyles, poor dietary habits rich in ultra-processed foods and low in essential nutrients, chronic stress, and exposure to environmental pollutants [1]. In recent years, growing evidence has highlighted another key player in the pathogenesis of these diseases: the gut microbiota and its balance, known as eubiosis [2].

The gut microbiota is a highly complex ecosystem composed of trillions of microorganisms, including bacteria, viruses, fungi, and archaea, which play a crucial role in regulating many physiological processes. These include digestion and nutrient absorption, energy metabolism, immune modulation, and the production of bioactive metabolites with both local and systemic effects [3]. Alterations in the composition and function of this microbial community, referred to as dysbiosis, have been linked to low-grade chronic inflammation, increased intestinal permeability, and metabolic dysfunctions, all of which contribute to the development and progression of cardiovascular diseases, diabetes, obesity, and neurodegenerative disorders [4].

Among the main factors capable of modulating gut microbiota, diet plays a central role. Several studies have shown that diets high in refined sugars, saturated fats, and processed foods promote the proliferation of pro-inflammatory bacterial species while reducing microbial diversity, negatively impacting systemic health [5]. Conversely, dietary patterns rich in fiber, polyphenols, and unsaturated fats, such as the Mediterranean diet (MD), have been associated with a more diverse and health-promoting microbiota [6].

The MD is characterized by a predominant intake of plant-based foods (fruits, vegetables, legumes, whole grains, nuts, and seeds), the abundant use of extra virgin olive oil (EVOO), a moderate consumption of fish, dairy products, and red wine, and a limited intake of red meat and ultra-processed products [7]. This dietary pattern has been extensively studied for its beneficial effects on human health [8,9,10,11,12]. Numerous epidemiological studies and clinical trials have demonstrated that high adherence to the MD is associated with a reduced risk of cardiovascular diseases, improved glycemic control, lower incidence of obesity, and slower cognitive decline [13,14,15]. The effectiveness of this dietary model appears to derive not only from its individual components but also from their synergistic interaction and their ability to influence the gut microbiota [16].

Emerging evidence suggests that the MD promotes the growth of beneficial bacteria that produce bioactive metabolites, such as short-chain fatty acids (SCFA), while simultaneously reducing the abundance of microbial species associated with inflammation and metabolic dysfunction [17]. These effects contribute to improved gut barrier integrity, reduced intestinal permeability, and lower activation of systemic inflammatory pathways, which are key factors in the prevention of chronic diseases.

This review aims to explore the role of the MD as a modulator of gut microbiota composition and to analyze its protective effects against major chronic diseases. By reviewing the most recent scientific literature, we discuss the mechanisms through which this dietary pattern influences gut and systemic health, with a particular focus on studies supporting its effectiveness in preventing and managing cardiovascular, metabolic, and neurodegenerative diseases.

## 2. Main Components of the Mediterranean Diet

The MD is one of the most extensively studied dietary patterns due to its well-documented health benefits, particularly in preventing cardiovascular, metabolic, and neurodegenerative diseases. This dietary approach, deeply rooted in the traditions of the regions bordering the Mediterranean Sea, is characterized by a predominant consumption of plant-based foods, a moderate intake of animal-derived products, and the use of healthy fats, primarily EVOO [18]. One of the distinctive features of the MD is its high content of fruits and vegetables, whole grains, legumes, nuts, and fish, while limiting the consumption of red meat, refined sugars, and ultra-processed foods [19]. This combination of foods provides a balanced nutritional profile capable of modulating numerous physiological processes [20].

Numerous epidemiological, clinical, and interventional studies have documented the benefits of the MD, which translate into antioxidant, anti-inflammatory, and protective effects on the cardiovascular and metabolic systems [21,22,23]. Beyond its benefits for human health, the MD is also recognized as a sustainable dietary model [24]. The predominant use of plant-based foods contributes to reducing environmental impact by limiting greenhouse gas emissions, water consumption, and land use.

EVOO is the primary fat source of the MD (recommended dose 20–40 g/die). Obtained through mechanical processes, it stands out for its favorable lipid profile and high content of bioactive compounds [25,26,27,28]. Rich in monounsaturated fatty acids (MUFAs) [29], it contains 70–80% oleic acid, known for its cardioprotective properties [30]. This fatty acid modulates nuclear receptors PPARα and PPARγ, improving insulin sensitivity [31], and reduces LDL cholesterol levels by activating hepatic LDL receptors [32,33].

The PREDIMED study demonstrated that the MD enriched with EVOO or nuts reduces the risk of major cardiovascular events [34]. EVOO contains phenolic compounds such as oleuropein, hydroxytyrosol (HT), and tyrosol, which have antioxidant, anti-inflammatory, and antimicrobial activities (recommended dose 5–10 mg/die) [35,36]. Oleuropein inhibits NF-κB, reducing pro-inflammatory cytokines (IL-6, TNF-α) [37], stimulates NO production, improving vasodilation, and activates antioxidant enzymes that neutralize reactive oxygen species (ROS) [38].

HT protects membrane lipids and DNA from oxidative damage and regulates antioxidant defense enzymes [39]. Recent studies show that it reduces phosphatidylserine exposure on erythrocyte membranes, regulating their elimination through eryptosis [40,41,42], a process that, if excessive, promotes thrombus formation and vascular micro-occlusions [43,44]. For this reason, HT is being studied for cardiovascular disease prevention [45].

Oleuropein and HT also regulate tumor cell proliferation by modulating the cell cycle and inducing apoptosis [46,47]. They influence matrix metalloproteinases [48], reducing tumor cell invasiveness, and improve endothelial function and arterial stiffness, protecting cardiovascular health [49,50].

The antioxidants in EVOO modulate gut microbiota composition, generating systemic anti-inflammatory effects mediated by SCFA production [51,52,53]. EVOO’s benefits also extend to the brain: its phenolic compounds protect against cognitive decline and neurodegenerative diseases like Alzheimer’s [54,55].

Alongside EVOO, the MD promotes a high intake of fruits and vegetables, which serve as a primary source of dietary fiber, essential for gut health (recommended dose 400–800 g/die). Fiber improves intestinal motility and modulates glucose and lipid absorption, thus contributing to metabolic regulation [56]. Fruits and vegetables are rich in essential vitamins, including vitamin C (recommended dose 75–90 mg/die), a potent antioxidant and cofactor in collagen synthesis; vitamin A (recommended dose 700–900 μg/die), crucial for vision and immune health; and vitamin K (recommended dose 90–120 μg/die), essential for blood clotting and bone metabolism. Additionally, they provide B vitamins, which are fundamental for energy metabolism and nervous system function. These foods also contain high concentrations of essential minerals such as potassium, magnesium, and iron, which are necessary for electrolyte balance and cellular energy production. The presence of these minerals in the MD helps reduce blood pressure and improve cardiovascular function [57,58]. Lastly, fruits and vegetables are abundant in phytochemicals such as flavonoids, carotenoids, polyphenols, and glucosinolates, bioactive compounds with antioxidant and anti-inflammatory properties [59,60].

Among flavonoids, quercetin (recommended dose 10–50 mg/die), abundant in onions, apples, and broccoli, modulates the activation of JNK kinase and reduces oxidative stress through the activation of the Nrf2 transcription factor [61]. Catechin (recommended dose 150–300 mg/die), present in berries, inhibits lipid peroxidation and improves endothelial function, reducing arterial stiffness [62]. Carotenoids, such as beta-carotene (recommended dose 6–15 mg/die), a precursor of vitamin A, promote cell differentiation and reduce the expression of pro-inflammatory cytokines like IL-1β [63,64]. Lycopene, found in tomatoes, is a powerful ROS scavenger, protecting against oxidative DNA damage [65]. Finally, glucosinolates (recommended dose 20–100 mg/die), compounds found in cruciferous vegetables (broccoli, cabbage), are converted into isothiocyanates, which modulate the enzymatic activity of glutathione S-transferases, protecting cells from environmental carcinogens [66]. Regular consumption of fruits and vegetables is associated with a reduction in blood pressure and LDL cholesterol levels, thanks to the synergistic action of fiber, potassium, and antioxidants, contributing to the prevention of cardiovascular diseases.

Another pillar of the MD is the consumption of fish and seafood, valued not only for their flavor but also for their numerous nutritional benefits. Fatty fish, such as salmon, mackerel, sardines, and tuna, are among the primary sources of omega-3 fatty acids (recommended dose 250–500 mg/die), which play a crucial role in supporting the cardiovascular and nervous systems [67]. Additionally, fish provides high-biological-value proteins, containing all essential amino acids necessary for the maintenance and repair of body tissues. It is also a rich source of essential minerals: iodine, indispensable for thyroid hormone synthesis; selenium, a powerful antioxidant; and zinc, which supports immune function. Equally important are the vitamins provided by seafood products, including vitamin D, crucial for bone health and immune system regulation, and B vitamins. Epidemiological studies have shown that regular fish consumption reduces the risk of coronary heart disease and stroke. Omega-3 improve endothelial function, lower blood pressure, and prevent platelet aggregation [68].

Whole grains, such as oats, spelt, barley, buckwheat, and quinoa, represent a valuable source of nutrients with multiple health benefits (recommended dose 90–180 g/die). They are rich in complex carbohydrates, which provide long-term energy and help stabilize blood glucose levels. Additionally, due to their high dietary fiber content, they support gut health, promote weight control, and enhance satiety. These foods also provide powerful antioxidants such as phenolic acids, lignans, and phytosterols, which protect cells from oxidative stress. Dietary fibers promote the growth of gut microbiota and the production of SCFA, reducing systemic inflammation. Furthermore, they regulate glucose and lipid absorption, improve insulin sensitivity, and lower LDL cholesterol levels [69,70].

Legumes (beans, lentils, chickpeas) and nuts (almonds, walnuts, pistachios) also represent an essential component of the MD (recommended dose 15–30 g/die). Legumes provide high-quality plant proteins, serving as an excellent alternative to animal proteins. They contain unsaturated fats, such as oleic acid and linoleic acid, and a high content of both soluble and insoluble fiber. Phytosterols (recommended dose 200–400 mg/die), present in legumes and nuts, reduce cholesterol absorption at the intestinal level, contributing to cardiovascular health [71]. Additionally, pistachios contain polyphenols and carotenoids, such as lutein and zeaxanthin, which have antioxidant properties and may enhance gut health. Some studies suggest that these bioactive compounds also support cognitive function by reducing oxidative stress and inflammation. In particular, pistachios and peanuts have been shown to positively influence gut microbiota composition, increasing beneficial bacteria like *Lactobacillus* and *Bifidobacterium*, which contribute to improved metabolic health. Moreover, peanut consumption has been associated with a reduction in harmful bacteria, such as *Bilophila*, which are linked to inflammation and metabolic disorders [72]. The beneficial effects of regular consumption of legumes and nuts have also been widely documented in clinical settings. In particular, they help reduce cardiovascular risk by decreasing the incidence of heart attacks and strokes due to the combination of healthy fats, fiber, and phytosterols [73]. They are also useful in preventing type 2 diabetes, as plant proteins and fiber improve insulin sensitivity and help regulate blood glucose levels. Additionally, the bioactive compounds in these foods have anti-inflammatory effects, reducing systemic inflammatory markers such as C-reactive protein [74].

Red wine, when consumed in moderation, is a characteristic component of the MD, thanks to its high polyphenol content, including resveratrol, quercetin, catechins, and anthocyanins. Organic acids, such as tartaric and malic acid, regulate pH and improve the bioavailability of micronutrients, while flavonoids modulate crucial biological pathways for metabolic and cardiovascular health. Resveratrol, for example, activates sirtuins, enhancing cellular resistance to stress and promoting longevity [75]. Moreover, it has anti-angiogenic effects, inhibiting tumor growth by reducing VEGF expression, and protects the cardiovascular system by reducing LDL oxidation and improving endothelial function through increased NO bioavailability [76,77]. Quercetin, with its potent antioxidant activity, protects cells from free radical damage and suppresses inflammatory processes by inhibiting the NF-κB signaling pathway [78]. Catechins, finally, improve cardiovascular function, reduce arterial stiffness, and prevent platelet aggregation, as well as modulate the activity of endogenous antioxidant enzymes [79]. Epidemiological studies, such as the Lyon Diet Heart Study, have shown that moderate red wine consumption is associated with a reduced risk of cardiovascular events [80]. However, it is essential to emphasize that the benefits of red wine are observed only with moderate consumption, generally defined as one glass per day for women and two for men. Excessive consumption, on the other hand, can have negative health effects, including an increased risk of liver disease, cardiovascular problems, and alcohol dependence. Therefore, red wine can be considered a health ally only when incorporated in balance within a healthy lifestyle.

Finally, dairy products in the MD, such as yogurt and traditional cheeses, are often whole and produced through artisanal methods, which best preserve their nutritional value (recommended dose 30–40 g/die). These foods are an excellent source of high-biological-value proteins, thanks to the presence of casein and whey proteins, rich in essential amino acids. They also contain fats, predominantly saturated, with a significant fraction of SCFA. Dairy products are particularly rich in essential minerals such as calcium and phosphorus, fundamental for bone health, and some, such as yogurt and fermented cheeses, provide probiotics that promote a balanced gut microbiota composition, with positive effects on the immune system and inflammation reduction [81,82]. At the molecular level, the bioactive nutrients present in dairy products exert specific functions. Probiotics improve gut microbiota composition, reduce systemic inflammation, and promote SCFA production [83]. Calcium is not only crucial for muscle contraction, blood clotting, and intracellular signaling but also helps reduce saturated fat absorption in the intestine, supporting body weight control. Butyric acid promotes colon health by facilitating the elimination of abnormal cells and reducing intestinal inflammation [84]. The beneficial effects of regular dairy consumption are well documented. Regarding bone health, their supply of calcium, phosphorus, and vitamin D is associated with increased bone mineral density and a reduced risk of osteoporosis [85]. Additionally, bioactive peptides derived from casein may have hypotensive effects by inhibiting the angiotensin-converting enzyme, contributing to cardiovascular health [86].

**Figure 1:**
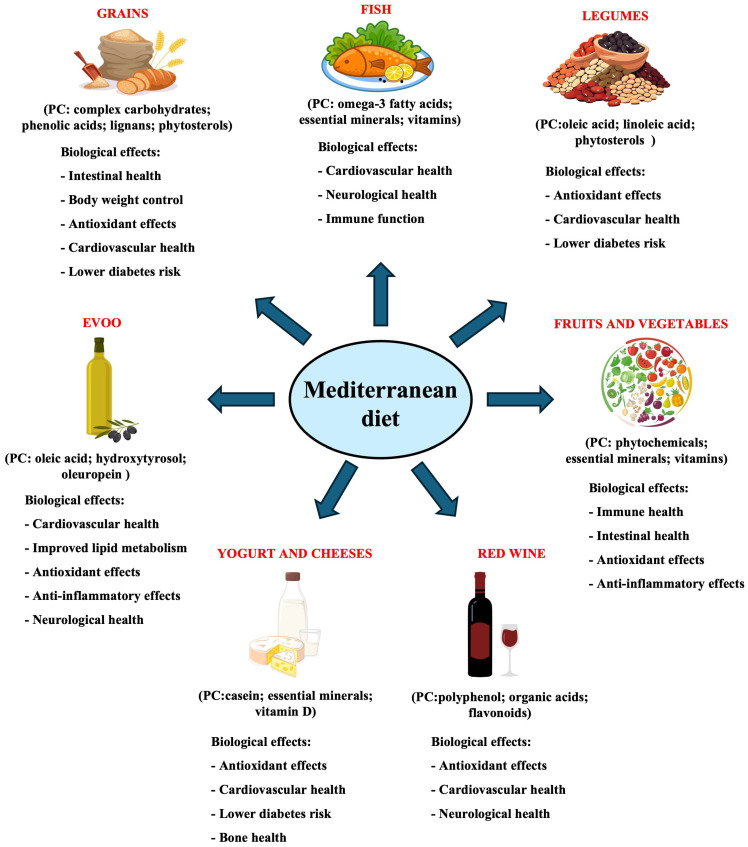
Main components of the MD with their principal effects on human health. PCs: principal components.

In summary, the MD stands out not only for its balanced nutritional profile but also for the positive impact it has been shown to exert on systemic inflammation and metabolic markers (Figure 1) [87]. It is important to note that, in recent years, research has increasingly focused on the connection between the MD and the gut microbiota. Recent studies suggest that the MD not only promotes a more diverse and beneficial microbial composition but may also modulate the microbiota in a way that enhances overall well-being and reduces the risk of diseases associated with dysbiosis. Exploring this relationship, which is gaining increasing relevance in the scientific community, is essential for better understanding the mechanisms through which the MD can act as a tool for prevention and therapy.

## 3. Intestinal Microbiota

The intestinal microbiota represents one of the most complex and fascinating microbial ecosystems within the human body. This community of microorganisms, which lives in symbiosis with the human organism, is a fundamental element for health and well-being, influencing a multitude of physiological processes [88]. Its composition, functions, and the pathological implications associated with its alterations are the subject of intense scientific studies due to its crucial impact on various aspects of human physiology. This microbial ecosystem, composed of trillions of microorganisms, was first systematically described in the 1960s through early classical microbiology studies. However, it was only with the advent of next-generation sequencing technologies and the application of metagenomic approaches, starting in the early 2000s, that the complexity and extraordinary diversity of the intestinal microbiota were unveiled. The term “microbiota” refers to the collection of all microorganisms residing in the human intestine, while “microbiome” indicates the entire collective genetic heritage of this community. The intestinal microbiota develops immediately after birth and continues to mature throughout childhood, influenced by genetic, environmental, and dietary factors [89]. It represents an intricate and dynamic ecological network that establishes a symbiotic relationship with the human host, contributing not only to digestion and metabolism but also to immune system modulation, metabolic homeostasis regulation, and bidirectional communication with the central nervous system [90].

The composition of the microbiota varies based on genetic factors, diet, age, environment, and the use of medications such as antibiotics. Under normal conditions, a balance exists among the various microbial species, helping to maintain intestinal homeostasis. This balance is known as eubiosis, whereas dysbiosis is a condition of microbial imbalance associated with numerous diseases [91]. From the early years of life, the intestinal microbiota begins to colonize the gastrointestinal tract. This process is influenced by numerous factors, including the mode of delivery (natural birth or cesarean section), diet (breastfeeding or formula feeding), environment, and exposure to microorganisms. During the first thousand days of life, the microbiota develops rapidly, undergoing significant transformations that impact long-term health [92].

Studies have shown that infants born through natural delivery acquire an initial microbial flora similar to the maternal vaginal microbiota, while those born via cesarean section develop a microbiota more similar to that of the maternal skin. This difference may have implications for susceptibility to infections and allergies [93]. During childhood and adolescence, the microbiota continues to evolve, influenced by diet and the gradual introduction of solid foods. The transition from a milk-based diet to a diversified diet leads to significant changes in microbial composition, with an increase in bacterial species capable of degrading complex carbohydrates and producing beneficial metabolites such as SCFA [94]. In adulthood, the microbiota reaches relative stability, although it remains susceptible to environmental factors, diet, stress, and pharmacological treatments, particularly the use of antibiotics [95].

The intestinal microbiota is an extraordinarily diverse microbial community composed of bacteria, archaea, viruses (including bacteriophages), and fungi (Table 1). The human gut hosts approximately 10^13^–10^14^ microorganisms, a number that exceeds that of human cells in the body, with an estimated total weight of about 1–2 kg. Among its main components, bacteria represent the predominant fraction of the intestinal microbiota. Among the approximately 50 described bacterial phyla, the most abundant are *Firmicutes* and *Bacteroidetes*, which together constitute over 90% of the intestinal bacterial population. Other present phyla include *Actinobacteria*, *Proteobacteria*, *Fusobacteria*, and *Verrucomicrobia*. *Firmicutes* include genera such as *Lactobacillus*, *Clostridium*, and *Faecalibacterium*, which are involved in the fermentation of complex carbohydrates and the production of SCFA. *Bacteroidetes*, with genera such as *Bacteroides* and *Prevotella*, play a crucial role in fiber degradation and immune system modulation. The balance between *Firmicutes* and *Bacteroidetes* (F/B ratio) is often used as an indicator of intestinal and metabolic health [96,97]. Archaea, although present in smaller quantities compared to bacteria, play a significant role in regulating intestinal gases. *Methanobrevibacter smithii*, the dominant archaeon, contributes to methanogenesis, a process that reduces hydrogen accumulation produced during microbial fermentation. The intestinal virome includes both viruses that infect eukaryotic cells and bacteriophages, which regulate bacterial population dynamics. Bacteriophages, in particular, modulate microbiota diversity and stability through predation mechanisms and horizontal gene transfer. Fungi represent a minor but significant component of the intestinal microbiota. Genera such as *Candida*, *Saccharomyces*, and *Malassezia* are involved in digestion and immune response; however, excessive fungal growth is often associated with dysbiosis and inflammation [98]. Some commensal protozoa, such as *Blastocystis*, have recently been recognized as potential regulators of intestinal health, although their role remains a subject of scientific debate [99]. The composition of the microbiota varies along the gastrointestinal tract. In the stomach, the highly acidic environment limits microbial density, favoring the presence of acid-tolerant bacteria such as Helicobacter pylori. In the small intestine, there is a moderate microbial diversity, with bacteria involved in the digestion of simple nutrients and the regulation of peristalsis. Finally, the colon hosts the majority of the intestinal microbiota, with a microbial density of approximately 10^12^ cells/mL [97]. In this region, fiber fermentation and the production of bioactive metabolites take place.

One of the most fascinating aspects of the microbiota is its multifunctional role [100]. In particular, the intestinal microbiota is essential for numerous physiological processes, which can be categorized into three main functions: metabolic, immunological, and protective [90]. Among its metabolic functions, the microbiota is responsible for digesting complex, non-digestible compounds such as dietary fibers and oligosaccharides, producing SCFA like acetate, propionate, and butyrate. These metabolites provide energy for enterocytes and exert anti-inflammatory effects. Additionally, the microbiota contributes to the synthesis of essential vitamins, including vitamin K and certain B vitamins, and plays a role in lipid metabolism and energy balance, influencing fat accumulation. Regarding immunological functions, the microbiota stimulates the maturation of the immune system and helps maintain a balance between immune tolerance and response. For instance, bacteria such as *Bacteroides fragilis* promote the production of regulatory T lymphocytes, which suppress inflammation [101]. Furthermore, the microbiota regulates the production of cytokines and antimicrobial peptides by intestinal epithelial cells. The protective functions include forming a barrier against pathogens through competition for nutrients and space. The microbiota also produces antimicrobial substances, such as bacteriocins, which inhibit the growth of pathogenic microorganisms [102]. Finally, recent studies have shown that the microbiota can communicate with the central nervous system through the gut–brain axis, using neurochemical signals and metabolites such as SCFA and tryptophan, influencing neurological and behavioral functions, including mood and stress response [103].

The importance of the intestinal microbiota has been corroborated by numerous studies highlighting its role in various pathological conditions, including obesity, type 2 diabetes, and chronic inflammatory bowel diseases. Additionally, microbiota alterations have been linked to a wide range of disorders affecting not only the gastrointestinal tract but also other organs and systems, such as neuropsychiatric disorders and cardiovascular diseases [104]. In the context of gastrointestinal diseases, irritable bowel syndrome (IBS) represents a key example. Dysbiosis has been frequently observed in IBS patients, characterized by reduced microbial diversity and an increase in pro-inflammatory species. Altered SCFA production and microbiota-driven immune modulation contribute to symptoms such as abdominal pain, diarrhea, or constipation [105]. Another significant example is chronic inflammatory bowel diseases (IBD), such as Crohn’s disease and ulcerative colitis. Studies have shown that IBD patients often exhibit a reduction in beneficial bacteria like *Firmicutes* and an increase in pathogenic bacteria. This imbalance promotes chronic inflammatory responses, exacerbating symptoms and disease progression [106]. Among the most severe intestinal infections are *Clostridioides difficile* infections, often triggered by antibiotic use that disrupts microbial balance. This infection is associated with severe diarrhea and pseudomembranous colitis [107].

Microbiota alterations are not confined to the gastrointestinal tract but have significant effects on metabolism. For instance, in obesity and type 2 diabetes, the microbiota can influence energy metabolism, promoting fat accumulation and insulin resistance. An increased *Firmicutes/Bacteroidetes* ratio has often been observed in obese individuals [108]. Another metabolic disorder associated with dysbiosis is non-alcoholic fatty liver disease. Dysbiosis can promote hepatic lipogenesis through the production of metabolites such as SCFA and affect intestinal permeability, contributing to liver inflammation [109].

As previously described, the implications of the microbiota also extend to neurological health. Dysbiosis, for example, has been associated with mood disorders such as depression and anxiety via the gut–brain axis. Alterations in neurotransmitter production, such as serotonin, and changes in systemic inflammatory responses are among the involved mechanisms. Furthermore, emerging studies suggest a possible involvement of the microbiota in neurodegeneration, as seen in Alzheimer’s and Parkinson’s diseases. In these cases, chronic inflammation and the production of neurotoxic metabolites appear to play a crucial role [103]. Autoimmune diseases represent another area where the microbiota appears to play a crucial role. For instance, in type 1 diabetes, early-life dysbiosis has been associated with an increased risk of developing the disease, likely due to immune system dysregulation. Another autoimmune condition influenced by the microbiota is multiple sclerosis, where microbial alterations can modulate immune responses both systemically and within the central nervous system [110].

To address the consequences of dysbiosis, several therapeutic strategies have been developed to modulate the microbiota. A diet rich in fiber, prebiotics, and probiotics promotes a healthy microbiota by increasing microbial diversity and enhancing the presence of beneficial species such as *Bifidobacterium* and *Lactobacillus*. Conversely, diets high in saturated fats, sugars, and processed foods can encourage the proliferation of pathogenic species, contributing to dysbiosis. Probiotics, composed of live microorganisms, can help restore microbial balance and improve gut health. Prebiotics, on the other hand, are non-digestible substances that stimulate the growth of beneficial bacteria. One of the most innovative therapies is fecal microbiota transplantation, which involves transferring microbiota from a healthy donor to a patient to restore microbial diversity. This procedure has proven particularly effective in treating *Clostridioides difficile* infections [111]. Personalized diets and microbiota-targeted drugs are also emerging as promising strategies. For example, fiber-rich and fermented food diets can promote a healthy microbiota, while new drugs are being designed to influence specific microbiota components.

**Figure 2:**
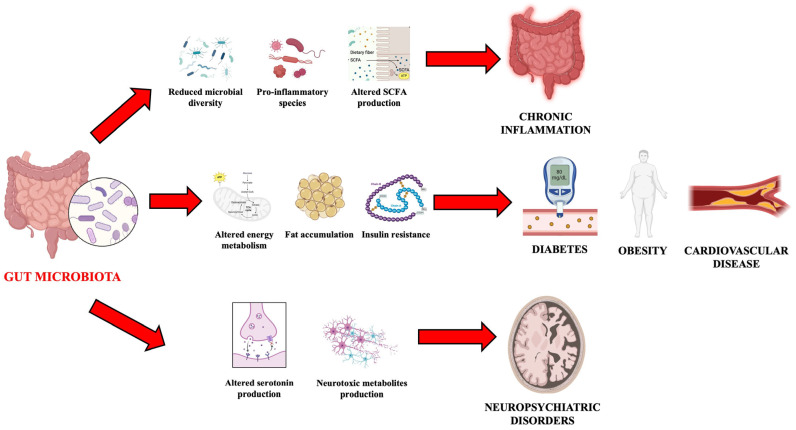
Major diseases associated with alterations in the gut microbiota.

The intestinal microbiota is an essential component of human physiology, influencing a vast range of biological processes. The increasing understanding of its functions and interactions with the host is opening new therapeutic perspectives for numerous diseases (Figure 2). Promoting microbiota health through targeted interventions represents a crucial strategy for improving long-term health and well-being.

Based on the above findings, the importance of proper nutrition in maintaining microbiota health is evident. Consequently, in recent years, several studies have highlighted the potential of the MD in preventing intestinal dysbiosis due to its richness in fiber, antioxidants, and unsaturated fatty acids, which support microbial diversity and the maintenance of eubiosis.

However, the variety of approaches taken and the variability of results necessitate a systematic review to analyze and integrate the currently available evidence. This would allow for a better understanding of the effectiveness and mechanisms by which the MD can be used as a preventive and therapeutic tool in maintaining intestinal microbiota health.

## 4. Methodology

A narrative review of the literature on the effect of MD supplementation in improving human microbiota health was conducted. The databases consulted included SCOPUS, Google Scholar, and PubMed (MEDLINE). Articles were selected based on title, year of publication (between 2009 and 2024), review of abstracts, and reading the full text to assess relevance. The keywords used in the search included a combination of terms: “Mediterranean diet”, “gut microbiota”, “olive oil”, and “polyphenols”. Articles without access to the full text were not included in the final analysis. Studies were included and selected according to their relevance in associating improved health related to microbiota with the use of the MD in animal and human models.

## 5. Effect of Mediterranean Diet on Gut Microbiota

The scientific literature offers a wide range of often heterogeneous results regarding the effect of MD on gut microbiota (Table 2). Several studies have analyzed how the MD alters the intestinal microbial composition and how these changes may impact metabolic health, inflammation, and even cognitive decline. The interaction between diet and gut microbiota could partially explain the effectiveness of the MD in preventing or managing metabolic and neurodegenerative diseases. The dietary modifications characteristic of the MD plays a crucial role in modulating gut microbiota composition. These changes promote an increase in SCFA production, reduce systemic inflammation, and have beneficial effects on various aspects of health [18]. Additionally, the consumption of polyphenol-rich foods and the use of EVOO appear to have a synergistic effect in improving microbial composition, contributing to gut and metabolic health [51].

**Effect of MD in mouse models:** Numerous preclinical studies have explored the role of gut microbiota as a mediator of MD effects, focusing on fatty acids, polyphenols, and dietary fibers using murine models. For example, it has been demonstrated that an 8-week high-fat diet (HFD) profoundly alters gut microbiota in mice fed a high-fat diet. This leads to intestinal barrier impairment, increased systemic inflammation, and the development of obesity and insulin resistance through the JNK/IRS (Ser 307) pathway [112]. However, the administration of HT reversed these effects by improving intestinal barrier integrity and restoring microbial balance. Furthermore, the protective effects of HT have been observed to be transferable through fecal transplantation. A study on spontaneously hypertensive rats fed an EVOO-enriched diet for 12 weeks showed an increase in gut microbial diversity and a reduction in systolic blood pressure. These results were associated with an increase in beneficial bacteria, such as *Clostridia* cluster XIV, confirming the link between EVOO and microbiota modulation [113]. These findings have been further confirmed by other studies exploring the use of EVOO as a modulator of gut microbiota [114]. The study in question examined the impact of a diet enriched with EVOO or butter on the gut microbiota of mice, correlating the results with physiological and biochemical parameters associated with metabolic syndrome. EVOO was found to promote greater gut microbial diversity compared to the use of refined oils. This microbiota modulation may be linked to improvements in physiological and biochemical parameters related to metabolic syndrome.

**Effect of the MD on microbiota composition:** An area of great interest is the connection between the MD, microbial composition, and SCFA. SCFA are known for their anti-inflammatory properties and their role in strengthening the intestinal barrier, reducing gut permeability, and improving metabolic health. De Filippis et al., through a metagenomic analysis, observed that greater adherence to the MD in a sample of 153 Italian individuals led to an increase in SCFA-producing bacteria, such as *Roseburia* and *Bifidobacterium*, both beneficial for gut health. The authors highlight the importance of a significant intake of plant fibers, polyphenols, and EVOO in promoting the growth of these bacteria. Additionally, participants with lower adherence to the MD showed higher levels of trimethylamine N-oxide, a metabolite associated with cardiovascular diseases, further confirming that an altered microbiota composition may be linked to a higher risk of metabolic diseases [115].

A parallel randomized study conducted by Meslier et al. examined the effect of the MD in a group of obese subjects. After an 8-week intervention, researchers observed a significant increase in the genetic richness of the gut microbiome, associated with reduced systemic inflammation and improved metabolic parameters, including insulin sensitivity. Adherence to the MD led to an increased prevalence of *Akkermansia muciniphila* and *Faecalibacterium prausnitzii*, bacteria known for their role in protecting against chronic inflammation and improving lipid metabolism [116]. A further study by Garcia-Mantrana et al. [117] examined how dietary habits influence microbiota composition, demonstrating that greater adherence to the MD was associated with a higher abundance of *Bifidobacterium* and an increased production of SCFA, such as acetate. Specifically, dietary information from 27 healthy volunteers was recorded using a food frequency questionnaire, while adherence to the MD was measured using the PREDIMED test. The results of this study suggest that a high intake of plant-based foods, including plant proteins and polysaccharides, promotes a healthier microbial composition characterized by a higher prevalence of SCFA-producing bacteria, with positive effects on gut and metabolic health. In fact, a significantly higher presence of *Christensenellaceae* was found in individuals with normal weight compared to those who were overweight. Higher bifidobacteria counts and total SCFA levels were correlated with greater consumption of plant-derived nutrients, such as plant proteins and polysaccharides. Furthermore, a well-balanced MD was associated with a lower presence of pro-inflammatory bacteria, such as *Escherichia coli*, suggesting a protective role against the development of gut dysbiosis and inflammatory diseases.

Gut microbiota diversity is recognized as a key indicator of gut itself health. In particular, the complex interactions between genes and the environment are considered important in the development of obesity. The composition of the gut microbiota can determine the efficiency of energy extraction from food, and dietary composition changes have been linked to shifts in intestinal microbial populations. Several studies have highlighted how MD can increase microbiota diversity. Specifically, Cotillard et al. [118] demonstrated that a diet rich in fiber, polyphenols, and monounsaturated fatty acids, characteristic of the MD, enhances gut microbiota diversity. A high level of microbial diversity was associated with a reduction in the prevalence of pro-inflammatory and pathogenic bacteria, as well as a decrease in circulating inflammatory markers such as C-reactive protein (CRP). Wu et al. [119] also explored the effect of the MD on the distribution of microbial enterotypes, identifying an enterotype dominated by *Bacteroides*, which was associated with better nutrient absorption and more efficient regulation of lipid metabolism. In particular, the authors showed that plant fibers and polyphenols appear to promote the growth of SCFA-producing bacteria and improve gut health while reducing bacterial toxin production and preventing dysbiosis.

**Cardiovascular diseases:** Another area of research concerns the effect of the MD on weight management and cardiovascular risk factors. A recent study of 2024 analyzed the impact of a calorie-restricted MD intervention combined with physical activity in 400 participants aged 55 to 75, half of whom were at high risk for cardiovascular diseases [120]. The results showed that the intervention group experienced greater weight loss and improvements in cardiovascular risk factors, such as blood pressure and cholesterol levels. Additionally, changes in the fecal microbiota were observed, with an increase in microbial diversity and a reduction in the abundance of genera such as *Eubacterium hallii* and *Dorea*, which have been associated with poorer metabolic health. These microbiota changes correlated with modifications in fecal metabolite profiles, including bile acids and ceramides, which were linked to improvements in cardiovascular risk factors. These findings suggest that the MD, in combination with physical activity, can promote a microbial composition that helps manage weight and reduce cardiovascular disease risk.

**Obesity and metabolic dysfunction:** Similarly, Haro et al. [121] demonstrated that MD consumption can restore gut microbiota dysbiosis in obese patients, depending on the degree of metabolic dysfunction. The authors analyzed bacterial community differences at baseline and after two years of dietary intervention in 106 study participants, including 33 obese patients with severe metabolic disease, 32 obese patients without metabolic dysfunction, and 41 non-obese subjects. The dietary pattern increased microbial diversity while reducing obesity-associated pathogenic bacteria (e.g., *Firmicutes*). There was a significant increase in beneficial bacteria such as *Bacteroides* and *Lactobacillus*. Additionally, reductions in fasting glucose and insulin levels, along with a marked decrease in pro-inflammatory cytokines, suggested an anti-inflammatory effect of the diet. The results indicate that long-term MD consumption partially restores gut microbiota dysbiosis in obese patients with coronary artery disease, depending on the degree of metabolic dysfunction.

A recent clinical study further highlighted that the MD improves metabolic profiles and modulates gut microbiota, particularly in individuals with obesity and metabolic syndrome [122]. The authors hypothesized that the MD may influence gut microbiota through alterations in vitamin D levels. During a one-year intervention, adherence to a calorie-restricted MD led to increased microbial diversity in participants with best vitamin D levels, along with significant changes in bacterial taxa associated with key metabolites, such as *Bacteroides* and *Prevotella*. Both study groups reported improvements in serum vitamin D levels and microbiota diversity, suggesting that lifestyle and diet can work synergistically to modulate gut microbiota and metabolic pathways. Furthermore, the findings show an intriguing association between vitamin D status and the composition, diversity, and functionality of gut microbiota. Lifestyle interventions thus remain an effective means to simultaneously influence microbiota composition and vitamin D levels, with potential benefits for metabolic pathways.

Interestingly, other researchers have explored the hypothesis that gut microbiome composition plays a key role in metabolic differences among individuals with different body weights, even under similar genetic conditions, such as in twins. The study by Turnbaugh et al. [123] analyzed fecal samples from pairs of monozygotic and dizygotic twins, where one sibling was obese. The microbiota samples were transplanted into germ-free mice to determine whether microbiota from obese or lean individuals could influence body weight and metabolism in recipient mice. The results showed that the gut microbiome of lean twins had greater microbial diversity compared to that of obese twins. In particular, lean individuals’ microbiota exhibited a higher abundance of bacteria associated with beneficial metabolic functions, such as *Bacteroidetes*. When microbiota from obese and lean twins was transplanted into germ-free mice, the animals that received the microbiota from obese twins gained more weight and had greater fat deposition than those that received microbiota from lean twins, despite following an identical diet. This suggests that differences in gut microbiota can significantly impact energy storage capacity and metabolism. Additionally, researchers observed that the microbiota from lean twins was associated with higher production of beneficial metabolites and a more effective regulation of metabolic pathways involved in energy consumption. These findings indicate that lean individuals’ microbiota has a superior ability to protect against fat accumulation by modulating both inflammation and energy balance. This study highlights the potential of gut microbiota and the use of the MD to modulate its composition as a therapeutic target for combating obesity and metabolic diseases.

**Neurological disorders:** The influence of the MD on gut microbiota has also been explored in relation to aging and cognitive health. Ghosh et al. [124] followed 612 elderly individuals across five European countries for one year, observing that adherence to the MD was associated with improved cognitive function and reduced frailty. The gut microbiota composition of individuals following the MD showed an increase in *Bacteroidetes* and *Firmicutes* taxa. Notably, changes in microbiome composition correlated with increased SCFA production and improved cognitive markers. These results align with the study by Nagpal et al. [125], which investigated the effect of the MD on the gut microbiome of individuals with mild cognitive impairment. In this study, the MD was shown to modulate gut microbiota in a way that improved Alzheimer’s biomarkers in cerebrospinal fluid, suggesting that the MD may have positive effects on brain health through gut microbiome modulation.

**Gastrointestinal diseases:** At the gastrointestinal health level, adopting the MD has shown significant effects. In a 2017 study, adherence to the MD was associated with increased bowel movement frequency and more pronounced gastrointestinal symptoms. Participants with higher adherence to the MD had a gut microbiota characterized by a greater abundance of *Bifidobacterium* and a better balance between *Bifidobacterium* and *Escherichia coli*, suggesting that a diet rich in plant-based foods and polyphenols may improve gut microbial composition and promote healthy digestion. Additionally, higher total SCFA levels, particularly acetate, were correlated with better gut health and a lower incidence of gastrointestinal disorders [126].

**AIDS:** Another study investigated the effect of a MD enriched with EVOO and nuts on individuals with HIV, a population known to have an altered microbiota. After 12 weeks, subjects following the MD showed an improved lipid profile and reduced immune activation. Moreover, microbial diversity and richness significantly increased, with a rise in beneficial bacteria such as *Bifidobacterium*. These changes correlated with improved immune function, suggesting that the MD can positively modulate microbiota even in severe pathological conditions [127].

**Differences between the MD and other diets:** A more recent research focus has compared the MD with different diet types about microbiota health. The MD was evaluated against fast-food (FF) and vegetarian (VD) diets. An FF diet reduced fiber-fermenting bacteria (e.g., *Lachnospiraceae*) while increasing taxa associated with inflammation, such as *Bilophila wadsworthia*. Conversely, the MD enhanced SCFA production and neuroprotective metabolites like indole-3-propionic acid, highlighting how dietary habits can rapidly influence microbiota and its metabolites [128]. Compared to the VD, the MD influenced specific taxa and SCFA production differently, suggesting that both diets can modulate the microbiota but with distinct effects depending on dietary components and intervention duration [129].

Another study applied multi-omics approaches to investigate the effects of the MD compared to non-MDs. Significant findings included variations in circulating metabolites, such as lipids and amino acids, linked to improved insulin sensitivity. These changes were mediated by bacteria like *Faecalibacterium prausnitzii* and their metabolic products, which positively influenced energy metabolism and systemic inflammation [130].

Finally, several research groups have shown that MD variants can serve as innovative approaches to improving overall health concerning the gut microbiota. The Green-MD variant, which emphasizes plant-based foods such as *Mankai* and green tea, has shown even more pronounced effects than the traditional MD. In a study on individuals with abdominal obesity, the Green-MD diet improved microbiota composition by increasing taxa such as *Prevotella* and reducing *Bifidobacterium*, contributing to greater weight loss and improved cardiometabolic markers [131]. These findings suggest that alterations in gut microbiota composition may mediate the effects of the Green-MD diet.

Adding probiotics to the MD further enhanced microbiota diversity, with beneficial effects on blood glucose levels and insulin resistance in breast cancer survivors [132]. Similarly, a MD variant enriched with dairy products altered the abundance of taxa such as *Butyricicoccus*, improving blood pressure and glucose metabolism [133].

## 6. Discussion

The results of the studies reviewed confirm the importance of the MD as a modulator of gut microbiota, with positive effects on metabolic, cardiovascular, and cognitive health. Although individual responses may vary, adherence to the MD promotes a favorable microbial composition, improving gut health and modulating pathological processes related to chronic inflammation, metabolic syndrome, and neurodegenerative diseases (Table 3).

The key factors characterizing the MD include a high intake of plant fibers, monounsaturated fats, polyphenols, and the predominant use of EVOO, all of which positively influence gut microbiota composition [130]. Increased intake of soluble fibers stimulates the growth of SCFA-producing bacteria such as *Bifidobacterium*, *Roseburia*, and *Faecalibacterium prausnitzii*, which inhibit inflammation and promote intestinal barrier integrity. Additionally, the MD reduces the levels of pro-inflammatory bacteria such as *Escherichia coli*, which are associated with gut dysbiosis and inflammatory diseases [126]. This protective effect is particularly relevant for individuals with metabolic disorders, such as obesity and metabolic syndrome, and is evident through increased microbial diversity and a reduction in pathogenic bacteria, as observed in studies on obese and sedentary individuals after eight weeks of MD adherence.

Another crucial aspect is the role of polyphenols, natural compounds abundantly present in olive oil, red wine, fruits, and vegetables. Polyphenols are known to promote the growth of beneficial bacteria and have antioxidant effects, further contributing to the reduction of inflammatory burden. Studies have shown that regular consumption of EVOO improves gut microbial diversity, reduces the risk of cardiovascular diseases, and enhances metabolic parameters such as insulin sensitivity. Additionally, preclinical studies have highlighted the potential of olive oil to reverse the negative effects of high-fat diets on gut microbiota and systemic inflammation [52,114].

MD also exerts a protective effect on metabolic and cardiovascular diseases, partly mediated by gut microbiota. Studies such as those by De Filippis et al. and Meslier et al. have demonstrated a clear link between MD adherence, reduced systemic inflammation markers, improved metabolic profiles, and an increase in SCFA-producing bacteria [115,116]. Moreover, the integration of the MD with specific approaches, such as the addition of probiotics or the adoption of the Green-MD variant, has shown additional benefits in terms of microbial composition and cardiometabolic parameters [131].

Another relevant aspect concerns the relationship between gut microbiota and cognitive health. Recent studies suggest that the MD may positively influence cognitive decline, with microbiota modulating the production of neuroprotective metabolites, improving brain function, and potentially counteracting neurodegenerative diseases such as Alzheimer’s [125]. The MD promotes the growth of *Akkermansia muciniphila*, a bacterium that enhances metabolic function, gut health, and modulates brain inflammation.

Additionally, the MD appears to be beneficial for weight management. Studies have shown that in overweight individuals, adopting the MD leads to a significant reduction in obesity-related pathogenic bacteria, such as *Firmicutes*, and an increase in beneficial bacteria like *Lactobacillus*, which support digestion and fat metabolism. These changes in microbiota composition have been associated with physiological improvements, such as reduced blood glucose and insulin levels, as well as a decrease in pro-inflammatory cytokines, suggesting an anti-inflammatory effect that supports the management of obesity and related conditions [112,122].

Data from twin studies and murine models reinforce the idea that the gut microbiome plays a causal role in metabolic differences between individuals, even under similar genetic conditions [123]. The MD thus appears as a powerful tool to favorably modulate microbiota, reducing inflammation and improving energy balance. The gut microbiota, as demonstrated in animal models, influences the body’s response to diet, determining nutrient energy extraction and overall energy balance. The effects of the MD on microbiota extend beyond the gut, with implications for systemic and metabolic health, including the reduction of intestinal permeability, a mechanism associated with autoimmune and inflammatory diseases.

An underexplored aspect concerns the link between the MD and oral microbiota. The boundaries between the effects of gut and oral microbiota are not well defined, and recent studies suggest that the MD may also influence oral microbiome dysbiosis. Dysbiotic oral microbiota has been associated with numerous systemic pathologies, including metabolic and neurodegenerative disorders such as genetic Alzheimer’s disease [134,135]. Some studies have highlighted that a diet rich in polyphenols, such as the MD, can positively modulate the oral microbiome, reducing the proliferation of pathogenic bacteria involved in periodontitis and neuroinflammation [136]. This suggests that the beneficial effect of the MD may extend beyond gut microbiota, positively influencing oral health and reducing the risk of systemic diseases related to oral microbiota dysbiosis.

## 7. Conclusions

In summary, the MD represents a powerful tool for modulating gut microbiota, with positive effects on metabolic, cardiovascular, and cognitive health. The combination of fibers, healthy fats, polyphenols, and plant-based oils plays a crucial role in promoting the growth of beneficial gut bacteria. These compounds improve gut function, reduce inflammation, and regulate metabolism. Clinical and preclinical studies confirm that adherence to the MD can reduce the risks of cardiovascular disease, diabetes, and obesity while improving key parameters associated with these conditions.

The influence of the MD on cognitive health and brain aging opens new therapeutic perspectives, suggesting that gut microbiota may serve as an effective target for nutritional interventions aimed at preventing neurodegenerative diseases. Furthermore, the growing evidence that microbiota and its metabolites can be manipulated through diet supports the concept of personalized medicine, where targeted dietary modifications can optimize gut health and prevent systemic disorders linked to inflammation and chronic diseases.

However, certain areas require further exploration, particularly the interindividual variability in microbiota responses, which is influenced by genetic and environmental factors. While many studies are observational or short-term, larger and long-term clinical trials are necessary to fully understand the impact of the MD across different populations and health conditions.

## Figures and Tables

**Table 1:** Composition of the human microbiota.

Group	Genus/Species	Quantity (%)	Main Location
**Firmicutes**	*Lactobacillus*	~64%	Small intestine, colon
	*Clostridium*		Colon
	*Faecalibacterium*		Colon
**Bacteroidetes**	*Bacteroides*	~23%	Colon
	*Prevotella*		Colon
**Actinobacteria**	*Bifidobacterium*	~3%	Small intestine, colon
**Proteobacteria**	*Escherichia coli*	Variable	Small intestine, colon
	*Helicobacter pylori*	Low	Stomach
**Fusobacteria**	*Fusobacterium*	~2%	Colon
**Verrucomicrobia**	*Akkermansia muciniphila*	~2%	Colon
**Archaea**	*Methanobrevibacter smithii*	<1%	Colon
**Fungi**	*Candida*	<1%	Small intestine, colon
	*Saccharomyces*	<1%	Small intestine, colon
**Protozoa**	*Blastocystis*	Variable	Colon
**Viruses**	*Bacteriophages*	Variable	Small intestine, colon

**Table 2:** Comparison of experimental models associated pathologies and key observed beneficial factors of the main scientific papers describing the effect of the Mediterranean diet on the gut microbiota.

Model System	Associated Pathology	Key Beneficial Factors Observed	Reference
HFD mice	Obesity, inflammation, insulin resistance	HT improves gut barrier integrity and reduces inflammation	[112]
Hypertensive rats	Hypertension	EVOO increases microbial diversity and lowers blood pressure	[113]
Mice	Metabolic syndrome	EVOO promotes beneficial bacteria and reduces metabolic syndrome	[114]
153 Italian individuals	Metabolic health and inflammation	MD increases SCFA-producing bacteria and lowers TMAO	[115]
Obese subjects	Systemic inflammation and metabolism	MD increases *Akkermansia muciniphila* and reduces inflammation	[116]
27 healthy volunteers	Gut microbiota composition	MD increases *Bifidobacterium* and SCFA production	[117]
Clinical studies on humans	Inflammation and metabolism	Higher microbial diversity and reduction of pro-inflammatory bacteria	[118]
Clinical studies on humans	Lipid metabolism regulation	Favors SCFA-producing bacteria and reduces bacterial toxins	[119]
400 participants (ages 55–75)	Cardiovascular diseases and metabolism	Weight loss and improvement in cardiovascular risk factors	[120]
106 obese subjects	Obesity and gut dysbiosis	Increase in *Bacteroides* and *Lactobacillus*, reduction in pathogenic bacteria	[121]
Individuals with obesity and metabolic syndrome	Obesity and metabolic syndrome	Metabolic improvement and increase in vitamin D	[122]
Monozygotic and dizygotic twins	Metabolism and energy extraction	Greater microbial diversity in lean individuals compared to obese	[123]
612 elderly Europeans	Cognition and aging	Increase in SCFA and improved cognitive function	[124]
Subjects with mild cognitive impairment	Cognitive decline and Alzheimer’s	Microbiota modulation and reduction of Alzheimer’s biomarkers	[125]
Humans with gastrointestinal symptoms	Gut health	Better balance between *Bifidobacterium* and *Escherichia coli*	[126]
Individuals with HIV	Microbiota and immune system	Increased microbial diversity and improved immune function	[127]
Comparison of diets (FF vs. MD)	Inflammation and dysbiosis	Increase in SCFA and reduction in pro-inflammatory bacteria	[128]
Comparison of MD vs. vegetarian diet	Gut microbiota modulation	Distinct effects of MD and vegetarian diet on microbial diversity	[129]
Multi-omics study on MD patients	Insulin sensitivity and metabolism	Improved insulin sensitivity and energy metabolism	[130]
Subjects with abdominal obesity	Abdominal obesity and microbiota	Green-MED increases beneficial taxa and reduces inflammation	[131]
Cancer survivors (breast cancer)	Insulin resistance and blood glucose	Probiotics with MD improve blood glucose and insulin resistance	[132]
Subjects on the MD integrated with dairy	Blood pressure and glucose metabolism	Modifies bacterial taxa and improves blood pressure	[133]

**Table 3:** Effects of the MD on the microbiota and various related dysbiosis and association with different human diseases.

Effect of MD	Microorganisms Affected	Associated Dysbiosis	Related Human Diseases
**Increased SCFA production**	*Roseburia*, *Bifidobacterium*, *Faecalibacterium*	Reduced SCFA-producing bacteria	Metabolic syndrome, inflammation, obesity
**Improved gut barrier function**	*Akkermansia muciniphila*	Increased gut permeability	Irritable bowel syndrome (IBS), inflammatory bowel disease (IBD)
**Reduced systemic inflammation**	*Bacteroides*, *Lactobacillus*, *Christensenellaceae*	Higher levels of pro-inflammatory bacteria (*E. coli*)	Cardiovascular diseases, metabolic dysfunction
**Enhanced microbial diversity**	*Clostridia* cluster XIV, *Bacteroides*	Loss of microbial diversity	Obesity, metabolic syndrome, cognitive decline
**Modulation of lipid metabolism**	*Bacteroides*, *Faecalibacterium*	Disrupted bile acid metabolism	Cardiovascular diseases, obesity
**Lower TMAO levels**	*Prevotella*, *Bacteroides*	Higher levels of TMAO-producing bacteria	Cardiovascular diseases
**Reduction of pathogenic bacteria**	*Bifidobacterium*, *Lactobacillus*	Overgrowth of *Bilophila wadsworthia*, *E. coli*	Gut inflammation, metabolic disorders
**Improved insulin sensitivity**	*Akkermansia muciniphila*, *Bifidobacterium*	Gut microbiota imbalance	Type 2 diabetes, obesity
**Neuroprotective effects**	*Bacteroidetes*, *Firmicutes*	Dysbiosis affecting neurotransmitter production	Alzheimer’s, Parkinson’s, depression
**Weight loss and metabolic health improvement**	*Bacteroides*, *Lactobacillus*	Gut microbiota imbalance	Obesity, metabolic syndrome
**Increased beneficial bacteria in cardiovascular health**	*Faecalibacterium prausnitzii*, *Bifidobacterium*	Reduction in beneficial SCFA-producing bacteria	Hypertension, cardiovascular diseases
**Reduced gut inflammation in HIV patients**	*Bifidobacterium*, *Lactobacillus*	Microbial dysbiosis due to immune suppression	Gut inflammation in HIV patients

## Abbreviations

CAT	Catalase
EVOO	Extra virgin olive oil
FF	Fast-food diet
HT	Hydroxytyrosol
IBD	Inflammatory bowel disease
IBS	Irritable bowel syndrome
MD	Mediterranean diet
MUFA	Monounsaturated fatty acid
NO	Nitric oxide
ROS	Reactive oxygen species
SCFA	Short-chain fatty acid
VD	Vegetarian diet
